# Dental Decay and Filling Materials Considered in Their Electrical Relations

**Published:** 1878-07

**Authors:** S. B. Palmer

**Affiliations:** Syracuse, New York.


### ARTICLE II.

### *Dental Decay and Filling Materials Considered in their Electrical Relations.*

BY S. B. PALMER, M. D. S., SYRACUSE, NEW YORK.

*Mr. President, and Gentlemen of the Odontological Society.*—Having had the honor of election to associate membership in your Society, and being permitted to take part in your deliberations and share your hospitalities, it gives me great pleasure to present to you the following thoughts and deductions, as a contribution to the fund of general professional knowledge.

In presenting the conclusions to which my investigations have led me, I desire to say that, like the experiments upon which they are founded, they are independent of all parties, sides, or "departures," as such; for I believe that from the moment truth is sought on only one side of any question, investigation in that case becomes untrustworthy. Still, I wish to subscribe to certain new doctrines which have, of late years, become public, since I know that they spring from researches conducted in the true spirit of science, and appear to me to be the legitimate conclusions to be derived from undeniable facts.

There seems to be a considerable opposition to these doctrines in the profession, which mainly, so far as I can see, arises from the fact that ideas have been developed and reduced to practice through the agency of materials not heretofore considered as of the greatest utility or beauty. Yet the results of these ideas are too important to be overlooked, or derided. The idea seems to prevail that our new theories are founded upon the use of materials of an order too low to ensure such results as are claimed for them; and consequently, as it seems to me, opposition has been established without proper examination of the subject, and with still less effort to apply those materials where they are calculated to do the most good, namely, to remedy or abolish the failures incident to the best gold practice. It is to correct this state of misapprehension that I invite attention to those causes which render gold unreliable for use in chalky or poorly-calcified teeth.

At our meeting in November last there were presented ten "articles of faith," each of which comprehended much more of value than could be brought out in any single discussion. The result was, that the whole matter could not but be insufficiently considered, in the space of one meeting. As a text, therefore, for this paper, let me take the first article of that creed.

"Gold is the *best* material with which to save teeth." As I understand, this statement—bold, unqualified, dogmatic as it is—is copied from our text-books. I do not think the profession, as a rule, endorses this dictum. I rather believe that, to meet general views, it should be modified as follows: "Gold is the best material for fillings in cases where it is not incompatible with tooth-bone." Now, as opposed to this, we have the first article of the new creed: "In proportion as teeth *need* saving, gold is the *worst* material to use." This statement cannot be considered so sweeping as the one before quoted, for its very basis is an extremely important qualification. Moreover, while the first is as much, or more, the result of inheritance as of investigation, the second has been evolved from a long series of practical experiments, and depends for its verification solely upon admitted scientific truth. As the two now stand, they represent, not a contradiction, but the extremes of theory and practice as regards gold.

Teeth in a normal condition remain sound so long as there is harmony between the constituent substances of the teeth and the fluid media to which they are exposed. Any change in the latter, particularly if it produces an acid quality, is manifested by a softening or wasting of the surfaces of the teeth. This is a simple and well understood form of chemical action, and operates, as has been said, upon the whole exposed dental surface.

A second form of deterioration of tooth substance, is productive of cavities, through local action upon definite portions of a tooth or teeth. This usually occurs while the teeth and fluids are in a normal condition, through the lodgment and retention of particles of food or extraneous matters, until reaction or fermentation occurs in such places. This may be termed local, as the first instance may be called general, chemical action.

Here let me introduce, as corresponding to this local action, a simple form of galvanic battery; since we cannot conceive of chemical activity, however slight, without including the idea of a galvanic current. A tooth attacked by caries at a given point is like a battery cell containing the positive element alone—say zinc and an acid. In such a battery chemical action would be slow, with no manifestation of a current. In the tooth, the action which at first was simple soon becomes complex. When the lime salts are dissolved, the remaining cartilaginous portion corresponds to the porous cell of a Daniell battery. This comparison will, I think, aid in solving the question of why a tooth, having begun to decay, will sometimes cease that action altogether.

It was stated, in the discussions of the November meeting, that electricity has little to do with promotion of dental caries, and that we must look to chemical action for the causes of decay. Unless we can have a better understanding of the relations of chemical and galvanic action than is implied by such remarks, further argument on this subject is useless. It is that relation which we must understand; for upon it hang all the principles and forces with which we have to contend. To illustrate my position on this question, let me use, as a figure, ordinary combustion in a furnace. The fire being lighted, the surrounding air is rarefied, and a current or draft is established. The consumption of fuel is determined by the supply of air. With a low chimney, and coal dust as fuel, little heat or power is developed. But, small as is this power, let it be applied to forcing more air upon the slow fire which created it, or upon other fires, and we see a great augmentation of power obtained. Now cavities in teeth, lined as they are with softened bone, well represent slow combustion. The undissolved portions resist decay partially. But introduce now any agent which will act more energetically, and this resistance is reduced to little or nothing, and decay progresses more rapidly.

Any material which will offer a perfect and complete protection from corroding agencies will effectually stop and prevent decay. In proportion as it lessens in the degrees of resistance, decay advances. This ratio of failure of resistance depends upon the capabilities of the material as a conductor of heat and electricity. Gutta-percha is almost a non-conductor of both.

Viewed as to extremes, there are two general principles on which decay is attempted to be arrested. One mechanical; the other chemical. The first is that which is by far the most generally employed, and is the only one recognized by many. It consists simply in the exclusion of fluids; for without moisture or fluid circulation there can be no chemical action. This principle involves the use of any filling material which will meet the given indications. The chemical principle is of use only where the mechanical fails to fulfill the requirements. From this it is apparent that we must know what the requirements are; and further, that we must learn what action is set up between various filling materials and tooth substance.

Let us construct three miniature batteries, consisting of three teeth with a cavity in each. The crown and enamel correspond to the cell, the dentine answering for the positive element. In this condition each battery has but one solid element and one pole. The softened bone attached to the sound walls offers resistance to the action of the oral fluids, and thus, in these bone batteries, the consumption of the positive element is slow. To put these batteries into active working order, it is necessary only to remove the resisting medium (excavate the cavity,) and supply the negative element in the form of poor and leaky fillings of gold, tin and poor amalgam. Now observe the results to be obtained from these batteries.

With batteries in action the current obtained is according to the differences of the elements. Thus, platinum gives better results with zinc than with copper, and with copper better than with tin.

In our tooth batteries, the gold element, according to both law and experiment, produces the strongest and most constant current, and therefore consumes fastest the positive element. This result is that which is desired in galvanic batteries for mechanical and other purposes, but is the very thing to be avoided in filling teeth. In fact, the introduction of any metallic filling, under the above conditions, excites chemical action which results in the loss of tooth substance, upon the principles abvoe set forth.

The effect of such fillings in exciting decay is the same as that of a platinum plate when lowered into its place in a battery jar. The metal in both cases forms one pole, around and upon which is gathered electricity opposite in nature to that simultaneously gathered at the other pole. Thus is established a current, which obeys the laws of the electrical phenomena as well in the tooth as in the quart jar. The degrees of the currents in the different batteries differ in the following particulars.

Gold gives the strongest current because it is the best conductor. Its current is the most constant because the metal does not oxidize or corrode. Poor amalgam at first acts in the same manner as gold, though in a less degree, because it is not so good a conductor; but, later, this poor amalgam becomes itself oxidized, and the current is then lessened. Also the metallic coloring matter thrown off by oxidation is received into the softened bone until the once positive element becomes neutral, or in the same electrical condition as the plug. This accounts for the endurance of the black teeth filled, long ago and even defectively, with this quality of amalgam. Tin is low in conducting power as compared with gold. It is, in fact, in nearly the same electrical condition as tooth substance. Therefore the slight current thus evolved is not sufficient to decompose saliva and render acid action continuous. On the contrary, the compound resulting from its oxidation does not destroy vitality and penetrate tooth substance, as does that formed by amalgam.

In conclusion, then, the assertion is made that a certain percentage of teeth which cannot be preserved with gold, can be preserved with tin. If facts support this assertion, we meet here a power which we cannot ignore in considering questions as to filling materials. I think it better to at once accept this fact and turn it to account than to argue against the inevitable. In its physical manipulation, gold has no equal as a filling material; but in its chemical properties it is wanting. I advocate the principle, and assert the fact, that gold and tin combined make a filling which meets the indications for beauty, and for preservation of tooth-substance. Our European friends have long since applied this principle, by rolling gold and tin foil together. Such fillings must preserve teeth, although in appearance and endurance they are no better than good amalgam. It is my practice to apply two or three thicknesses of tin foil to the softened walls of the cavity, filling the remainder of the opening with gold, as usual. While these fillings serve all the purposes of gold, the small proportion of tin does not produce discoloration. However, I

do not put in any claim for the discovery of a combination of tin and gold, in any form. I instituted my investigations for the purpose of ascertaining the action of filling materials upon the teeth. I am fully satisfied that my conclusions are supported by laws of nature which, at no distant day, will be known, recognized, and reduced to practice, in dentistry, to the improvement of the profession and the good of humanity.—*Dental and Oral Science Magazine.*

